# Effect of Drought Stress during Soybean R2–R6 Growth Stages on Sucrose Metabolism in Leaf and Seed

**DOI:** 10.3390/ijms21020618

**Published:** 2020-01-17

**Authors:** Yanli Du, Qiang Zhao, Liru Chen, Xingdong Yao, Huijun Zhang, Junjiang Wu, Futi Xie

**Affiliations:** 1 Soybean Research Institute, Shenyang Agricultural University, Shenyang 110866, China; dyl0305@sina.cn (Y.D.); zqiang0416@hotmail.com (Q.Z.); clr0301@sina.com (L.C.); xingdongyao@syau.edu.cn (X.Y.); Zhj20047@sina.com (H.Z.); 2 Soybean Research Institute of Heilongjiang Academy of Agricultural Sciences, Key Laboratory of Soybean Cultivation of Ministry of Agriculture P. R. China, Harbin 150086, China; nkywujj@126.com

**Keywords:** *Glycine max*, drought, source–sink relationship, sucrose metabolism, sucrose transporter

## Abstract

Sucrose is the main photosynthesis product of plants and the fundamental carbon skeleton monomer and energy supply for seed formation and development. Drought stress induces decreased photosynthetic carbon assimilation capacity, and seriously affects seed weight in soybean. However, little is known about the relationship between decreases in soybean seed yield and disruption of sucrose metabolism and transport balance in leaves and seeds during the reproductive stages of crop growth. Three soybean cultivars with similar growth periods, “Shennong17”, “Shennong8”, and “Shennong12”, were subjected to drought stress during reproductive growth for 45 days. Drought stress significantly reduced leaf photosynthetic rate, shoot biomass, and seed weight by 63.93, 33.53, and 41.65%, respectively. Drought stress increased soluble sugar contents, the activities of sucrose phosphate synthase, sucrose synthase, and acid invertase enzymes, and up-regulated the expression levels of *GmSPS1*, *GmSuSy2*, and *GmA-INV*, but decreased starch content by 15.13% in leaves. Drought stress decreased the contents of starch, fructose, and glucose in seeds during the late seed filling stages, while it induced sucrose accumulation, which resulted in a decreased hexose-to-sucrose ratio. In developing seeds, the activities of sucrose synthesis and degradation enzymes, the expression levels of genes related to metabolism, and the expression levels of sucrose transporter genes were enhanced during early seed development under drought stress; however, under prolonged drought stress, all of them decreased. These results demonstrated that drought stress enhances the capacity for unloading sucrose into seeds and activated sucrose metabolism during early seed development. At the middle and late seed filling stages, sucrose flow from leaves to seeds was diminished, and the balance of sucrose metabolism was impaired in seeds, resulting in seed mass reduction. The different regulation strategies in sucrose allocation, metabolism, and transport during different seed development stages may be one of the physiological mechanisms for soybean plants to resist drought stress.

## 1. Introduction

Soybean (*Glycine max* (L.) Merr.) is the main edible oil, edible protein, and feedstock crop grown globally, with 120 million hectares planted and around 352 million tons of annual production at present [1]. Soybean plant growth and yield are markedly reduced by various abiotic stresses [2]. Drought stress is one of the primary environmental stress conditions that decreases crop productivity and quality, thus posing a serious threat to agriculture [3]. During the seed filling stage, which is the key stage determining seed size, weight, and composition as well as final soybean yield, soybean plants are more sensitive to water deficits than during vegetative growth. Seed filling involves the processes of carbohydrate mobilization and transport as well as the biochemical synthesis of proteins and lipids in developing seeds [2,4]. Therefore, understanding the physiological and molecular mechanisms underlying soybean yield under drought stress during the filling stage benefits the improvement of seed yields, thereby increasing food security.

Sucrose, the main photosynthetic product of higher plants, is not only the carbon foundation of physiological metabolism, but also a signaling molecule that coordinates the relationship between plant sources and sinks, playing an important role in plant growth and seed development [5,6]. In higher plants, the phosphotriose produced by photosynthesis is transported to the cytoplasm and converted to sucrose by enzymes such as sucrose phosphate synthase (SPS) and sucrose phosphate phosphatase. SPS irreversibly catalyzes the formation of sucrose phosphate from uridine diphosphate glucose (UDPG) and fructose 6-phosphate [6]. Activity of SPS, as one of the key rate-limiting enzymes in sucrose synthesis, plays an important role in regulating the sucrose content of plant cells. For example, a decrease in SPS activity in *Arabidopsis thaliana* inhibited the synthesis of sucrose and resulted in decreased soluble sugar content [7]. Sucrose can be reversibly converted to fructose and UDP-glucose by sucrose synthase (SuSy) or irreversibly hydrolyzed into glucose and fructose by soluble invertases, including cytoplasmic neutral/alkaline invertase (NI) and acid invertase (AI) [5]. Previous studies have shown that the activity of these sucrose metabolic enzymes in plants is directly affected by abiotic stress. For example, drought stress increased SPS activity and decreased SuSy activity in rice plants [8] and decreased the activity of invertase in soybean pods [9]. The genes involved in SPS, SuSy, and invertases (INVs) also played important roles in responding to environmental stresses [10,11]. RNA sequencing analysis has shown that drought stress before maize tasseling can reduce the expression levels of cell wall invertase (*CWIN*), soluble invertase (*INV*), and neutral invertase *(CIN*) genes in maize ovaries [12]. The expression levels of *SuSy* and *SPS* in cotton leaves were up-regulated as water-logging was prolonged [13]. A more recent study demonstrated that the inhibition of assimilate distribution induced rice grain weights to decrease under heat stress, which was associated with impaired sugar allocation and prominent changes in the expression of sucrose synthase genes such as *OsSuSy2*, *OsSuSy3*, and *OsSuSy4* [14].

Seeds are an important sink organ in soybean plants, and their final qualitative and quantitative traits are determined by the seed filling process and nutrient reserve accumulation, which involve biochemical processes related to carbohydrate import and accumulation as well as protein and lipid synthesis [15], which are significantly affected by environmental conditions [5]. Seed filling in plants depends upon the directly transported sucrose produced by photosynthesis in leaves, and transport capacity and efficiency are associated with the final seed weight (i.e., seed size). At the same time, sink strength and various physiological metabolic activities in sink cells, especially their metabolic activities, involved in assimilate consumption and accumulation feedback onto and mediating sucrose transport from sources to sinks [6,16]. In general, sucrose is transported into sink organs by the phloem via the symplastic or apoplastic pathway [6,17,18]. For symplastic phloem loading, sucrose transport relies on plasmodesmata, while for apoplastic pathway loading or unloading, sucrose is required by sucrose transporter proteins, such as those in the SWEETs transporter family (SWEETs) and sucrose transporter (SUC), to enable efficient sucrose movement across membranes [19,20,21,22]. In *Arabidopsis* leaves, AtSWEET11 and AtSWEET12 (plasma-membrane Suc effluxers) [23] are responsible for secreting sucrose from mesophyll or phloem parenchyma cells into the apoplast. AtSUC2 (the companion cell Suc-specific Suc:H^+^ symporter) is responsible for loading sucrose into the phloem [24,25]. Previous research has shown that sucrose transporter proteins are affected by abiotic stress. For example, the expression levels of *AtSUC2* and *AtSUC4* genes in *Arabidopsis* were induced by various stresses, including salt, low temperatures, osmotic stress, and abscisic acid (ABA) treatment [26]. Durand et al. (2016) [27] reported that the transcript levels of *AtSWEET11*, *AtSWEET12*, and *AtSUC2* genes in *Arabidopsis* leaves were up-regulated by drought stress, resulting in enhanced sucrose export to roots. Furthermore, heat stress repressed the expression of *OsSUT1* in rice leaf, grain, and sheath tissues and limited the assimilate supply for grain development, thereby reducing yield [28]. However, the regulation and management of sucrose transporter proteins in soybean leaves and seeds under drought stress are not fully understood.

Drought stress inhibits the photosynthetic carbon assimilation ability of plants and seriously affects seed weight. Photosynthetic assimilates, such as sucrose, are the carbon backbone of energy metabolism and amino acid biosynthesis for seed growth and development. However, knowledge about the roles of sucrose allocation, metabolism, and transport during soybean development during the seed-filling stages under drought stress is very limited. Therefore, we hypothesized that (1) soybean plants respond to drought stress at the reproductive stage by regulating the balance of sucrose metabolism and transport in leaf and seed tissues and the response mechanisms differ across growth stages and (2) the disruption of metabolism and transport balance results in decreased soybean seed weight. In this study, three soybean cultivars were used to (1) investigate the capacity for assimilation and accumulation of photosynthetic products under drought stress, (2) investigate sucrose metabolism regulation in response to drought stress in soybean leaf tissue and developing seeds by enzyme activity assays and qRT-PCR analyses, and (3) determine the capacity of sucrose transport in soybean leaf tissue and developing seeds under drought stress by qRT-PCR analysis.

## 2. Results

### 2.1. Effect of Drought Stress on Photosynthesis Rate, Shoot Biomass, and Seed Weight

**Figure 1:**
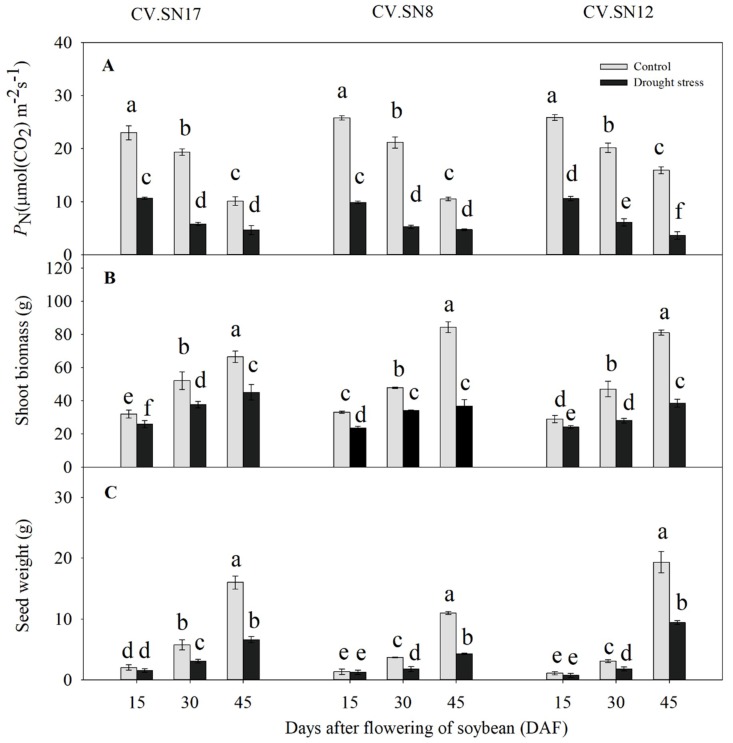
Effect of drought stress on photosynthesis rate, shoot biomass, and seed weight in different soybean cultivars. (**A**) The photosynthesis rates, (**B**) shoot biomass totals, and (**C**) seed weights of “Shennong17” (CV.SN17), “Shennong8” (CV.SN8), and “Shennong12” (CV.SN12). Standard deviations were calculated with three independent experiments each comprising two soybean plants. Different letters above vertical bars indicate significant differences between means at a *p* < 0.05 level.

Compared to the control, the net photosynthesis rate (*P*_N_) of the three examined soybean cultivars (“Shennong17” (CV.SN17), “Shennong8” (CV.SN8), and “Shennong12” (CV.SN12)) decreased by an average of 63.93% under drought stress during 15–45 days after flowering (DAF) (Figure 1A and Table S1). Furthermore, shoot biomass of the three soybean cultivars under drought stress had decreased by 19.77% at 15 DAF, 32.44% at 30 DAF, and 48.37% at 45 DAF, respectively, an average decrease of 33.53% (Figure 1B and Table S1). There were no significant differences in seed weight at the early seed development stage (15 DAF) between the control and drought stressed plants. However, seed weight decreased by 41.65% under drought stress compared to the control during the middle and late seed development stages (30–45 DAF) (Figure 1C). Compared with control, relative water content (RWC) of three soybean leaves was reduced by 10.73% under drought stress (Figure S1). Genotype (G), plant growth stage (S), treatment (T), and their interactions also significantly affected *P*_N_, shoot biomass, and seed weight (*p* < 0.05) according to a three-way ANOVA, except genotype for shoot biomass (Tables S1 and S2).

### 2.2. Effect of Drought Stress on Sugar Contents in Leaves and Developing Seeds

**Figure 2:**
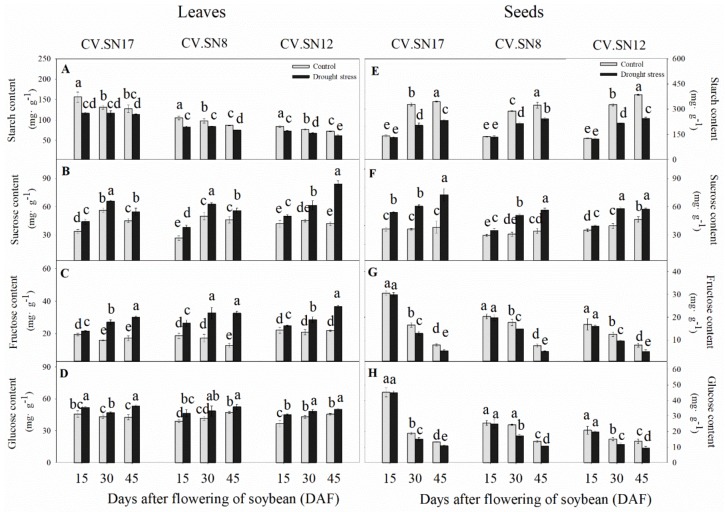
Effect of drought stress on the (**A**,**E**) starch, (**B**,**F**) sucrose, (**C**,**G**) fructose, and (**D**,**H**) glucose contents of leaves and seeds. Standard deviations were calculated with three independent experiments each comprising two soybean plants. Different letters above vertical bars indicate significant differences between means at a *p* < 0.05 level.

In leaves, drought stress significantly decreased the starch contents compared with control plants, with an average decrease of 15.13%. However, the sucrose, fructose, and glucose contents in leaves were significantly increased, with an average increase of 33.40, 61.59, and 15.50%, respectively, under drought stress compared to the control during 15–45 DAF (Figure 2A–D). In seeds, under control and stress conditions, the starch content increases, while the levels of fructose and glucose decrease during studied developmental stages (Figure 2E,G,H). In seeds of the three soybean cultivars, there were no significant differences in starch, fructose, or glucose content during early seed development stages (15 DAF) between the control and drought-stressed plants. However, drought stress significantly decreased the starch, fructose, and glucose contents of the three soybean cultivars during the middle and late seed development stages (30–45 DAF) (Figure 2E,G,H). Compared to the control, the sucrose contents of seeds under drought stress significantly increased by 48.24% during 15–45 DAF (Figure 2F). Three-way ANOVA revealed that genotype (G), plant growth stage (S), treatment (T), and their interactions also significantly affected the starch, sucrose, fructose, and glucose contents of leaves and seeds (*p* < 0.05), except the S × G interaction for starch and the S × T, G × T, and S × G × T interactions for glucose in leaves (Tables S1 and S2).

In leaves, drought stress significantly decreased the hexose-to-sucrose ratio of CV.SN17 and CV.SN8 at 15 DAF and CV.SN12 at 45 DAF by 13.01, 11.75, and 35.63%, respectively, compared to the control (Figure S2A). Meanwhile, compared to the control, the hexose-to-sucrose ratio in seeds of the three soybean cultivars were significantly decreased under drought stress during all reproductive stages (15–45 DAF) (Figure S2B).

### 2.3. Effect of Drought Stress on the Expression Levels of Sucrose Transporter Genes in Leaves and Developing Seeds

Sucrose is a major photosynthetic product and can be transported from sources to sinks in plants over long distances by sugar transporters (e.g., SWEET and SUC family proteins). To analyze the effect of drought stress on sucrose transport in soybean leaves and seeds, the expression levels of sucrose transporter genes were measured from the same samples used for sugar determination. Five soybean sucrose transporter genes, *GmSUC2* (homologous with *AtSUC2* from *A. thaliana*), *GmSWEET6*, *GmSWEET15*, and *GmSWEET12* (homologous with *AtSWEET11* and *AtSWEET12* from *A. thaliana*), and *GmSWEET21* (homologous with *AtSWEET10* from *A. thaliana*) [29] were selected for this study. We first evaluated the expression levels of all selected genes in CV.SN12 at 15 DAF in leaves and seeds under control conditions. Our results showed that *GmSWEET6* and *GmSWEET15* were highly expressed in leaves, while *GmSWEET12* and *GmSWEET21* were highly expressed in seeds (Figure S3), which was consistent with previous findings by Patil et al. [29]. Then, we further analyzed the expression levels of *GmSUC2*, *GmSWEET6*, and *GmSWEET15* in leaves and *GmSUC2*, *GmSWEET12*, and *GmSWEET21* genes in seeds under control and drought stress treatments.

**Figure 3:**
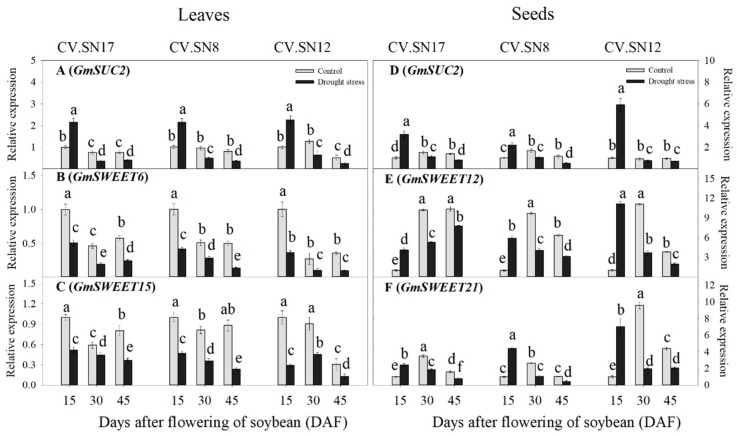
Effect of drought stress on the expression levels of sucrose transporter genes in leaves and seeds. (**A**–**C**) The expression levels of *GmSUC2*, *GmSWEET6*, and *GmSWEET15* in leaves. (**D**–**F**) The expression levels of *GmSUC2*, *GmSWEET12*, and *GmSWEET21* in seeds. Standard deviations were calculated with three independent experiments each comprising two soybean plants. Different letters above vertical bars indicate significant differences between means at a *p* < 0.05 level.

The qRT-PCR results revealed that drought stress significantly (*p* < 0.05) increased *GmSUC2* transcript levels in the leaves at 15 DAF (2.18-fold on average) and up-regulated the transcript levels of *GmSUC2*, *GmSWEET12*, and *GmSWEET21* in seeds (by 3.76-, 7.04- and 4.61-fold, respectively). However, under prolonged drought stress (i.e., 30–45 DAF), the transcript levels of *GmSUC2* in leaves and *GmSUC2*, *GmSWEET12*, and *GmSWEET21* in seeds were significantly (*p* < 0.05) down-regulated. In addition, drought stress significantly down-regulated the transcript levels of *GmSWEET6* and *GmSWEET15* in leaves during 15 to 45 DAF (Figure 3A–F, Tables S1 and S2). Genotype (G), plant growth stage (S), treatment (T), and their interactions also significantly affected expression levels of sucrose transporter genes (*p* < 0.05) in leaves and seeds according to a three-way ANOVA (Tables S1 and S2).

### 2.4. Effect of Drought Stress on Sugar Metabolism–Related Enzyme Activities in Leaves and Developing Seeds

**Figure 4:**
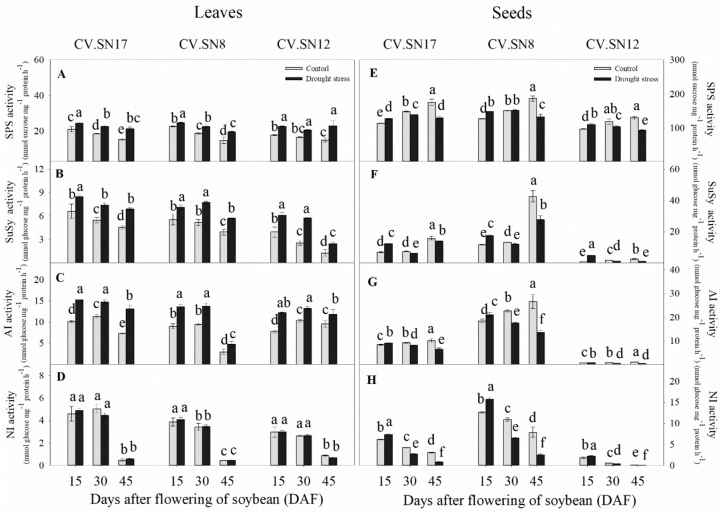
Effect of drought stress on the enzyme activity levels of (**A**,**E**) sucrose phosphate synthase (SPS), (**B**,**F**) sucrose synthase (SuSy), (**C**,**G**) acid invertase (AI), and (**D**,**H**) neutral/alkaline invertase (NI) in leaves and seeds. Standard deviations were calculated with three independent experiments each comprising two soybean plants. Different letters above vertical bars indicate significant differences between means at a *p* < 0.05 level.

To get a more insight on the effect of drought stress on sucrose utilization and partitioning between leaves and developing seeds, the activities of enzymes related to sucrose metabolism were analyzed. Drought treatment significantly increased SPS, SuSy, and AI enzyme activities in leaves during 15–45 DAF (Figure 4A–C, Table S1). However, there were no significant differences between the NI activities of control and drought-stressed leaves during 15–45 DAF (Figure 4D, Table S1). In seeds, the activities of SPS, SuSy, AI, and NI enzymes were significantly increased at 15 DAF under drought stress compared with the control, while the activities of those four enzymes were all sharply decreased during 30–45 DAF (Figure 4E–H, Table S2). Additionally, the activities of these enzymes related to sucrose metabolism were much higher in seeds than in leaves, even under drought stress (Figure 4). Three-way ANOVA revealed that genotype (G), plant growth stage (S), treatment (T), and their interactions significantly affected SPS, SuSy, AI, and NI activities (*p* < 0.05) in leaves, except for S × G × T interactions for SPS activity, G × T interactions for SuSy activity, and T for NI activity (Table S1). In seeds, genotype (G), plant growth stage (S), treatment (T), and their interactions significantly affected SPS, SuSy, AI, and NI activities (*p* < 0.05) except for G × T interactions for SPS activity and S and S × G interactions for AI activity (Table S2).

### 2.5. Effect of Drought Stress on the Expression Levels of Key Genes Involved in Sugar Metabolism in Leaves and Developing Seeds

To better understand the mechanisms of sucrose synthesis and degradation metabolism in leaves and seeds under drought stress, a qRT-PCR expression analysis was performed on eight genes belonging to the following three groups:

1. Four genes encoding putative sucrose phosphate synthases, i.e., *GmSPS1*, *GmSPS2*, *GmSPS3*, and *GmSPS4*;
2. Two genes encoding putative sucrose synthases, i.e., *GmSuSy1* and *GmSuSy2*;
3. Two genes encoding putative invertases, i.e., *GmA-INV*, which encodes acid-invertase, and *GmC-INV*, which encodes cytosolic neutral/alkaline invertase.

#### 2.5.1. Expression Levels of *GmSPS1*, *GmSPS2*, *GmSPS3*, and *GmSPS4* in Leaves and Developing Seeds

**Figure 5:**
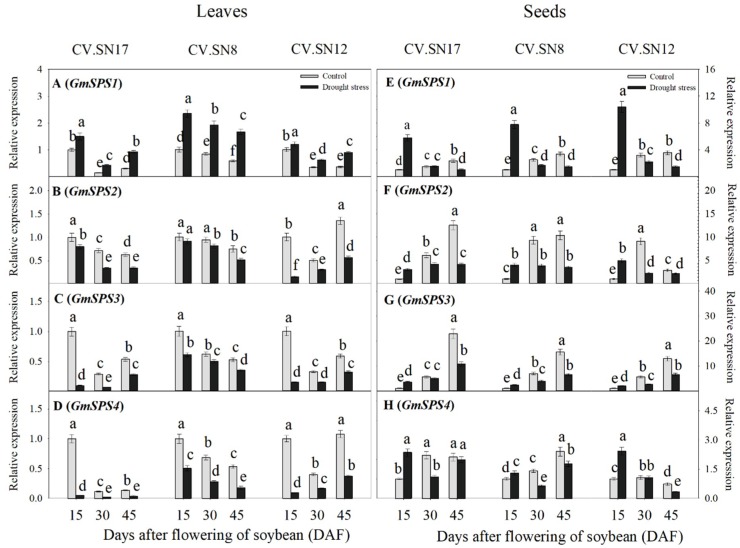
Effect of drought stress on the expression levels of *GmSPS* genes in leaves and seeds: (**A**,**E**) *GmSPS1*; (**B**,**F**) *GmSPS2*; (**C**,**G**) *GmSPS3*; and (**D**,**H**) *GmSPS4*. Standard deviations were calculated with three independent experiments each comprising two soybean plants. Different letters above vertical bars indicate significant differences between means at a *p* < 0.05 level.

The expression levels of four *GmSPS* genes in leaves showed different responses under drought stress (Figure 5A–F, Table S1). Drought stress significantly up-regulated the transcript levels of *GmSPS1* at 15–45 DAF, with an average 2.27-fold increase compared with the control. However, the expression levels of *GmSPS2*, *GmSPS3*, and *GmSPS4* were down-regulated during 15 to 45 DAF under drought stress compared to controls. In seeds, the expression levels of *GmSPS* genes under drought stress responded differently during different seed filling stages (Figure 5E–H, Table S2). Drought stress significantly promoted the expression levels of *GmSPS1*, *GmSPS2*, *GmSPS3*, and *GmSPS4* by averages of 7.97-, 3.99-, 2.60-, and 2.03-fold, respectively, at 15 DAF compared with control plants. However, transcripts of the four *GmSPS* genes were down-regulated during 30 to 45 DAF in drought-stressed plants, except that no significant differences between the control and drought stress treatments in *GmSPS4* transcript level were observed in CV.SN17 at 45 DAF and in CV.SN12 at 30 DAF, respectively (Figure 5E,F and Table S2). Three-way ANOVA revealed that G, S, and T, and their interactions also significantly affected the expression levels of *GmSPS1*, *GmSPS2*, *GmSPS3*, and *GmSPS4* (*p* < 0.05) in leaves and seeds, except T for *GmSPS4* in seeds (Tables S1 and S2).

#### 2.5.2. Expression Levels of *GmSusy1* and *GmSusy2* in Leaves and Developing Seeds

**Figure 6:**
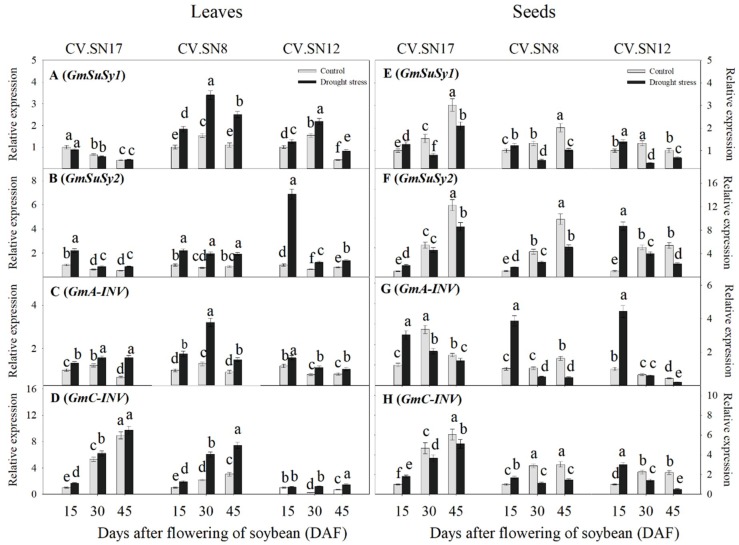
Effect of drought stress on the expression of (**A**,**E**) *GmSuSy1*; (**B**,**F**) *GmSuSy2*; (**C**,**G**) *GmA-INV*; and (**D**,**H**) *GmC-INV* in leaves and seeds. Standard deviations were calculated with three independent experiments each comprising two soybean plants. Different letters above vertical bars indicate significant differences between means at a *p* < 0.05 level.

In leaves, drought stress significantly up-regulated the transcript levels of *GmSusy1* during 15–45 DAF by an average of 2.11-fold in CV.SN8 and 1.57-fold in CV.SN12, respectively, relative to control plants. However, there were no significant differences in GmSusy1 transcript levels between the control and drought stress treatments in CV.SN17 during 15–45 DAF (Figure 6A and Table S1). The transcript levels of *GmSusy2* were up-regulated under drought stress in leaves during 15–45 DAF compared to the control (Figure 6B and Table S1). In seeds, the expression levels of *GmSusy1* and *GmSusy2* under drought stress responded differently across seed filling stages (Figure 6E,F, Table S2). Drought stress significantly up-regulated the expression levels of *GmSusy1* and *GmSusy2* at 15 DAF compared to control plants. However, *GmSuSy1* and *GmSuSy2* transcripts were sharply down-regulated in drought-stressed plants during 30 to 45 DAF (Figure 6E,F, Table S2). According to a three-way ANOVA, genotype (G), plant growth stage (S), and treatment (T), and their interactions significantly affected the expression levels of *GmSusy1* and *GmSusy2* (*p* < 0.05) in leaves and seeds, except G × T for *GmSusy1* in seeds (Tables S1 and S2).

#### 2.5.3. Expression Levels of *GmA-INV* and *GmC-INV* in Leaves and Developing Seeds

In leaves, drought stress significantly up-regulated the expression levels *GmA-INV* and *GmC-INV* during 15 to 45 DAF (Figure 6C and Table S1), except that there were no significant differences between the control and drought stress treatments in *GmC-INV* transcript level in CV.SN17 at 45 DAF and in CV.SN12 at 15 DAF, respectively. In seeds, the expression levels of *GmA-INV* and *GmC-INV* under drought stress responded differently across seed filling stages (Figure 6G,H and Table S2). Drought stress significantly up-regulated the expression levels of *GmA-INV* and *GmC-INV* at 15 DAF compared with control plants. However, *GmA-INV* and *GmC-INV* transcripts were all sharply down-regulated in drought-stressed seeds during 30 to 45 DAF (Figure 6G,H and Table S2). A three-way ANOVA revealed that genotype (G), plant growth stage (S), and treatment (T), and their interactions also significantly affected the expression levels of *GmA-INV* and *GmC-INV* (*p* < 0.05) in leaves and seeds (Tables S1 and S2).

## 3. Discussion

Many studies have shown that soybean growth, development, and yield are negatively affected by drought stress [9,30,31]. Drought stress inhibits the production of photosynthetic products owing to decreases in leaf photosynthetic capacity [32,33] and the acceleration of leaf wilting and senescence [2,34]. Drought stress during the reproductive phase, especially the seed filling stage, decreases the production and mobilization of assimilates to developing seeds, which can result in considerable yield losses [9,31]. In this study, drought stress during soybean seed filling stages significantly decreased *P*_N_, inhibited shoot growth, and decreased seed weight (Figure 1). Three soybean cultivars were used to study carbon assimilation, sucrose metabolism, and transport regulation mechanisms in response to drought stress during seed filling.

### 3.1. The Balance of Carbon Assimilation and Carbon Metabolism in Soybean Leaves was Disturbed by Drought Stress

**Figure 7:**
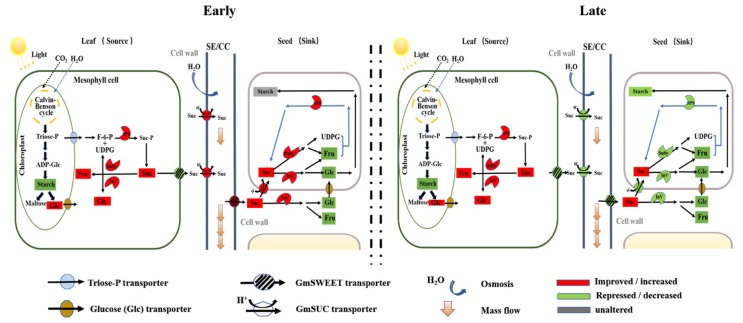
A comprehensive model of sucrose (Suc) metabolism and transport for sources (leaves) and sinks (seeds) in soybean plants under drought stress. Up-regulated elements under drought stress are shown with red boxes, down-regulated elements under drought stress are shown with green boxes, and unaltered elements are shown with gray boxes.

Drought stress significantly affected the distribution of carbohydrates in leaves (Figure 2 and Figure 7). This was consistent with previous research showing that drought stress decreased carbon assimilation while increasing accumulation of soluble carbohydrate content [27,35,36]. This regulation mechanism seemed to benefit plant growth and seed development, as higher sugar content of leaf tissue is responsible for sucrose phloem loading and flowing into sink organs, such as roots and seeds [37]. On the other hand, soluble sugars are important osmoprotectants and energy sources for plant cells under drought stress [38]. A previous study showed that the increased soluble sugars, mainly sucrose, improved plant tolerance to abiotic stress [39].

The accumulated soluble sugars might be related to drought stress–induced sugar metabolism regulation in soybean leaves. It was well known that SPS plays a vital role in recycling free UDPG and fructose to increase the sucrose content of leaves [6], while SuSy, AI, and NI are three important enzymes involved in sucrose decomposition [5,11]. The increased sucrose metabolic enzyme activities in soybean leaves under drought stress improved sucrose utilization efficiency and promoted soluble sugar accumulation (Figure 4A–D, Figure 5, and Figure 6). Notably, *GmSPS1* appeared to have a predominant role in sucrose synthesis in leaves compared to *GmSPS2*, *GmSPS3*, and *GmSPS4* under drought stress. This complex transcriptional regulatory mechanism of *GmSPS* genes in soybean leaves under drought stress requires further investigation.

Starch can be converted by triose phosphates in chloroplasts and stored as an energy source in leaves [40]. Thus, starch content is an important indicator of leaf source capacity. Drought stress induced a significant decrease in the starch content of soybean leaves (Figure 2A), which might be related to increased starch decomposition. Previous studies have shown that osmotic stress triggers starch degradation into glucose and maltose in leaves, leading to sugar accumulation [41,42]. It was apparent that the modification in partitioning between sucrose and starch in leaves occurred in response to drought stress.

### 3.2. Drought Stress Affects Sucrose Transport from Leaves to Seeds

Carbohydrates, mainly sucrose, are synthesized in mature source leaves and translocated to sink tissues (such as developing seeds) in higher plants to sustain heterotrophic metabolism and growth or for storage as sucrose or starch [43]. Previous studies have shown that the capacity of sucrose transport in higher plants is closely related to the expression of SWEET and SUC proteins in source and sink organs [29,44]. For example, Chen et al. (2012) [23] reported that a *sweet11/12* mutation in *A. thaliana* resulted in a reduction of sucrose excretion by leaves, and Dasgupta et al. (2014) [45] reported that up-regulation of *SUC2* increased [^14^C] sucrose uptake into the veins of leaf discs and higher phloem loading of sucrose. In the current study, drought stress regulated the expression of sucrose transport genes in soybean leaves and seeds at different seed development stages, causing (1) the inhibition of sucrose export in mesophyll cells, but the promotion of sucrose uptake by soybean seeds in early seed development, and (2) the inhibition of sucrose transport from leaves into seeds during later seed development (Figure 3A–F and Figure 7).

During early seed development, drought stress up-regulated the expression of *GmSUC2* in leaves and *GmSUC2*, *GmSWEET12*, and *GmSWEET21* in seeds (Figure 3A,D–F and Figure 7), which may promote more sucrose flowing into seeds to meet the energy demand for seed development [44,46,47], with minimum seed weight reduction occurring (Figure 1C). However, the down-regulated expression of *GmSWEET6* and *GmSWEET15* genes in soybean leaves during early seed development (Figure 4E,F) can induce sucrose accumulation in soybean mesophyll cells [23,29]. One reasonable explanation for the results is that the functions of *GmSWEET6/15* and *GmSUC2* proteins in leaves differ in response to early drought stress, as SWEET proteins are responsible for taking up sucrose from mesophyll cells or phloem parenchyma cells into the apoplast, and SUC proteins are responsible for loading sucrose into the phloem (Figure 7, [23,24,25]). The up-regulation of *GmSUC2* by drought stress might also mediate the ABA signaling pathway, as reported for *AtSUC2* in *Arabidopsis* [26]. The higher sugar content of leaves helped maintain mesophyll cell activity and the photosynthetic ability to respond to drought stress.

Two possible drought response mechanisms were proposed to explain the reduced expression levels of sucrose transport genes in leaves and seeds during 30–45 DAF: (1) drought stress created a signal that directly resulted in down-regulation of sucrose transport genes and the reduction of seed growth or (2) drought stress inhibited seed growth, thereby decreasing demand for sucrose in seeds, which exceeded the sucrose utilization capacity of seeds and, in turn, inhibited the expression of sucrose transport genes, thus ultimately reducing sucrose flow to seeds. Previous studies have shown that drought stress directly regulated expression levels of sucrose transport genes, such as *AtSWEET11*, *AtSWEET12*, and *AtSUC2*, thus mediating sucrose export from sources to sinks [27]. Water deficiency caused by drought stress in developing seeds can inhibit the synthesis and accumulation of seed reserves and block cell division [4,48], which might reduce energy demand and promote the early termination of the seed filling process. Thus, further experiments are needed to determine the mechanisms by which soybean sucrose transporter gene expression was affected by drought stress.

### 3.3. Drought Stress Affects Carbohydrate Enrichment and Metabolism in Developing Seeds

The seed filling period is the terminal stage for forming propagules in spermatophytes, and it involves several physiological and biochemical processes, such as the import of constituent molecules as well as carbohydrate, protein, and lipids synthesis in seeds [15]. These processes were significantly affected by various abiotic stresses and affected the final seed quality and weight [5,48]. When soybean plants were subjected to drought stress, seed starch content and seed weight were markedly decreased during the late seed filling stages (Figure 1C and Figure 2E) compared to the control, while sucrose concentration was significantly increased (Figure 2F). These results were similar to previous findings in lupin seeds when a short-term water deficit was imposed during seed development [34]. Previous studies have shown that the activities of sucrose synthase and soluble invertase decrease in drought-stressed seeds [9,34]. Interestingly, inconsistent with this previous conclusion, the activity levels of SPS, SuSy, NI, and AI also decreased in drought-stressed soybean seeds during the late drought-stress period (30–45 DAF) in our study; however, the activity levels of these four sucrose metabolism enzymes also increased during the early drought-stress period (15 DAF) (Figure 4E–H and Figure 7). Improved SPS activity can accelerate sucrose recycling and utilization [11]. SuSy, NI, and AI, which were also affected by drought stress, are three key enzymes involved in seed storage through their influence on sink activity [5,34]. Soybean plants were undergoing the beginning of seed formation during the early drought stress period (at 15 DAF), that is, sink growth needed more nutrition and starch accumulation during this period. Thus, increasing the activity levels of SPS, SuSy, NI, and AI enzymes was important for the improvement of drought resistance at the beginning of seed formation. This can be well verified by the constant contents of starch, fructose, and glucose in early drought-stressed developing seeds (Figure 2E–G), which contributed to maintaining seed weight (Figure 1C). However, during seed development and prolonged drought stress at 30–45 DAF, the activity levels of SPS enzymes decreased (Figure 4E and Figure 7) and sucrose re-synthesis was reduced, which suggests that sucrose recycling was limited. Meanwhile, the activity levels of SuSy, NI, and AI were decreased during 30–45 DAF (Figure 4F–H and Figure 7), suggesting that decomposition of sucrose was inhibited [5,49]. This was confirmed by the reduction of fructose and glucose content in drought-stressed seeds (Figure 2F,G). Previous studies have indicated that high hexose-to-sucrose ratios are favorable to cell division during reproductive development [5,9,50]. However, a decreased hexose-to-sucrose ratio was observed in drought-stressed seeds (Figure S2B), which suggests that seed cell division was inhibited by drought stress. Thus, the decline in sucrose degradation and recycling ability altered carbohydrate composition and allocation in seeds, which resulted in sucrose accumulation in seeds despite seed weight reduction (Figure 1C and Figure 2F). The change in expression of *GmSPS* genes in seeds of the three cultivars (Figure 5E–H) reflected the change in SPS activity (Figure 4E), which suggests that the four *GmSPS* homologs acted together to control sucrose synthesis in drought-stressed seeds. Previous studies have reported that loss-of-function invertase mutants had lower invertase activity and thus lower seed weights [51,52,53,54], which indicated that invertase activity had a critical role in the regulation of seed development. The role of *SuSy* gene was also important for carbon allocation during seed filling, especially when subjected to drought, cold, and heat stress [14,55]. In our study, a similar trend in expression levels among GmSuSy genes, *GmA-INV*, and *GmC-INV* (Figure 6E–H) reflected trends in SuSy, AI, and NI activity levels (Figure 4F–H) under drought stress. These results strongly suggest that *GmSuSy*, *GmA-INV*, and *GmC-INV* acted together to modulate sucrose degradation in developing seeds under drought stress.

## 4. Materials and Methods

### 4.1. Experimental Design and Treatment

Three soybean cultivars, “Shennong17” (CV.SN17, 20.77% oil (dry base) and 44.26% protein), “Shennong8” (CV.SN8, 20.40% oil and 42.89% protein), and “Shennong12” (CV.SN12, 22.09% oil and 38.89% protein), used in this research were obtained from the Soybean Institute, College of Agriculture, Shenyang Agricultural University, Liaoning, PR China. These soybean cultivars with similar growth periods are currently used in local production and no permission was needed for the collection of these materials. This pot cultivation experiment was conducted at the experimental station of Shenyang Agricultural University (41°82′ N, 123°57′ E) from May to September 2018. Average-sized soybean seeds were selected and planted directly into pots (25 × 30 × 25 cm) with 12.5 kg of soil per pot, and plants were then grown in a greenhouse. The soil (pH 7.33) contained 16.77 g/kg soil organic matter, 0.77 g/kg total nitrogen, 0.07 g/kg available nitrogen, 0.02 g/kg available phosphorus, and 0.14 g/kg available potassium. Experiments were conducted according to a randomized complete block design with three replicates. Each pot contained two plants undergoing the same treatment, which were together considered as a single experimental unit. The three soybean cultivars were planted in a total of 144 pots.

Drip irrigation was used to keep the soil moist at 70–80% of the field’s water-holding capacity until soybeans flowered. When about 50% of the flowers on the main stems of soybean were open (R2 growth stage, according to Fehr et al. (1971) [56]), different water treatments were applied to the soybean plants. Under treatment 1, normal watering (control) was conducted so as to maintain the relative soil water content at the field’s water holding capacity of 70–80%. Under treatment 2, a drought stress treatment was employed, that is, the relative water content was maintained at the field water holding capacity of 35–40%. Soil water levels were monitored using a soil moisture probe (Field Scout TDR 300 Probe, Spectrum Technologies, Inc., Aurora, IL, United States) every day. Ten pots were randomly selected for each treatment for soil water levels measurement, and the same pot was measured for three times. The first day after the drought stress treatment, i.e., the day after flowering (DAF), was recorded as 1 DAF, and the drought stress treatment lasted for 45 days. Tissue samples were taken at 15, 30, and 45 DAF, which represented the beginning of the seed formation phase (15 DAF, R4 growth stage), the start of the seed filling phase (30 DAF, R5 growth stage), and the rapid seed filling phase (45 DAF, R6 growth stage).

### 4.2. Sampling

To measure the shoot biomass and seed weight per plant, the aboveground parts of soybean plants were cut at the node of each plant’s cotyledon, oven-dried at 105 °C for 30 min, and maintained at 85 °C until the weight was constant. To measure leaf physiological and biochemical parameters, the third fully expanded leaf on each plant (from the top of the main stem) was selected at 9:30–11:00 and divided into two parts along its main veins; one half was immediately frozen in liquid N_2_ and stored at −80 °C for subsequent enzyme activity assays and gene expression analysis. The other half was dried at 105 °C for 30 min and oven-dried at 80 °C for the determination of starch, sucrose, glucose, and fructose contents.

Soybean seeds were also collected at 9:30–11:00 and similarly divided into two parts; one half was immediately frozen in liquid N_2_ and stored at −80 °C for subsequent enzyme activity assays and gene expression analysis. The other half was oven-dried for the determinations of starch, sucrose, glucose, and fructose contents.

### 4.3. Measurement of Net Photosynthetic Rate

To measure photosynthetic parameters, the third fully expanded leaf on each plant (from the top of the main stem) was selected at 9:30–11:00 and measured using a LI-6800 portable photosynthesis system (LI-COR Inc., Lincoln, NE, USA). According to the method described by Li et al. 2017 [57], the light-saturation point was set to 1200 μmol (photon) m^−2^ s^−1^ to measure the net photosynthetic rate (Figure S4). The ambient temperature of each measured soybean leaf was kept at 30 °C. The CO_2_ concentration was 400 μmol (CO_2_) mol^−1^, relative humidity was 60%–65%, and air flow was 500 μmol s^−1^.

### 4.4. Carbohydrate Measurement

Carbohydrates were extracted from leaves and seeds and then quantified using a modified version of the method described by Hu et al. 2018 [58]. About 0.1 g of ground sample was extracted with 80% (*v*/*v*) ethanol at 80 °C for 30 min, and then centrifuged at 10,000× *g* for 10 min at room temperature. The residue was extracted twice with 80% ethanol. The three supernatants were combined, and 80% ethanol was added to reach a total volume of 5 mL. Then, 20 μL extract samples were continuously incubated three times with glucose assay reagent (Glucose Assay kit; Sigma-Aldrich, St. Louis, MO, USA) at 30 °C for 15 min, with 10 μL of phosphoglucose isomerase (0.25 units, Sigma-Aldrich, St. Louis, MO, USA) at 30 °C for 15 min, and then with 10 μL of invertase (83 units, Sigma-Aldrich, St. Louis, MO, USA) at 30 °C for 60 min. The absorbance was recorded at A340 nm after each incubation step to determine glucose, fructose, and sucrose contents, respectively.

The ethanol-insoluble residue was used for starch extraction. After removing ethanol by evaporation, 2 mL of distilled water was added, and samples were incubated at 100 °C for 15 min. Then, 2 mL of 9.2 mol L^−1^ HClO_4_ was added into samples for 15 min to hydrolyze starch. Thereafter, 4 mL of distilled water was added to samples, which were then centrifuged at 4000× *g* for 10 min. The residue was extracted one more time using 2 mL of 4.6 mol L^−1^ HClO_4_. Then, the two supernatants were combined and distilled water was added to reach a total volume of 20 mL. According to the method by Kuai et al. 2014 [13], the starch content was determined by spectrophotometry using anthrone reagent at A620 nm wavelength.

Hexose concentration was calculated as the summation of fructose and glucose concentrations, from which hexose-to-sucrose ratios were calculated.

### 4.5. Enzyme Extraction and Analysis

According to Liu et al. 2013 [59], the frozen fresh samples were ground in liquid nitrogen and then homogenized with cooled extraction buffer containing 100 mM Tris-HCl (pH 7.2), 1 mM EDTA, 10 mM MgCl_2_, 10 mM β-mercaptoethanol, 10% polyvinylpyrrolidone (PVP), and 12.5% (*v*/*v*) glycerin. The homogenate was centrifuged at 15,000× *g* for 15 min at 4 °C. All extraction procedures were conducted at 0 °C–4 °C.

The reaction buffer for the SPS activity assay contained 12 mM UDP-glucose, 40 mM fructose-6-P, 200 mM Tris-HCl (pH 7.0), 40 mM MgCl_2_, and 200 μL of extract. For the SuSy activity assay, the reaction buffer contained 12 mM UDP, 40 mM sucrose, 200 mM Tris-HCl (pH 7.0), 40 mM MgCl_2_, and 200 μL of extract. The enzyme was incubated at 30 °C for 30 min to initiate reaction and 100 μL of 2 mol L^−1^ of NaOH was used to stop the reaction. Then, the solution was immediately heated to 100 °C for 10 min to destroy untreated hexose and hexose phosphates. After cooling the solution and adding 1 mL of 0.1% (*w*/*v*) resorcin in 95% (*v*/*v*) ethanol and 3.5 mL of 30% (*w*/*v*) HCl, the solution was incubated for 10 min at 80 °C. Sucrose content in the SPS reaction and fructose content in the SuSy reaction were calculated from a standard curve measured at A480 nm and A540 nm, respectively.

The neutral/alkaline invertase activities (NI) and soluble acid invertase (AI) were measured by incubating 100 μL of extract, 200 μL of 1 M sucrose, and either 2.2 mL of 100 mM sodium acetate–acetic acid (pH 7.5) (for alkaline invertase) or 2.2 mL of 200 mM acetic acid–NaOH (pH 5.0) (for acid invertase) for 30 min at 30 °C. The reaction was stopped with 1 mL of 3,5-dintrosalicylic acid (DNS) and boiling for 5 min. According to the method described by Hu et al. 2018 [59], glucose contents in AI and NI reactions were measured spectrophotometrically at A540 nm.

### 4.6. RNA Extraction and Complementary DNA Synthesis

Soybean leaf and seed tissues were harvested, and total RNA was isolated from each sample using a MiniBEST Universal RNA Extraction Kit (Takara, Kusatsu, Japan) according to the manufacturer’s protocol. RNA quantity and integrity were checked by optical density at 260 nm and 1.0% agar gel electrophoresis, respectively. Single-stranded cDNA was synthesized from 1 μg of total RNA using a PrimeScriptTM RT Reagent Kit (Perfect Real Time) (Takara, Kusatsu, Japan).

### 4.7. Gene Expression by qRT-PCR

For each sample, 1 μL of a reaction mixture was used for qRT-PCR in a 20 μL total reaction volume using TransScript^®^ Top Green qPCR SuperMix (TransScript, Beijing, China). Two reference genes, translation elongation factor 1α (*GmEF1a*) and translation elongation factor 1β (*GmEF1b*), were selected for examination in this study [60]. The following thermal cycle conditions were used: 95 °C for 1 min, followed by 39 cycles of 95 °C for 5 s, 58 °C for 20 s, and 60 °C for 20 s. The relative expression was determined according to the 2^−ΔΔCt^ method. The gene-specific primers are listed in Table S3.

### 4.8. Measurement of the Relative Water Content (RWC)

The fresh weight (FW) of soybean leaves was measured immediately after they were removed from the stem. Then, turgid weight (TW) was measured after leaves were incubated in distilled water for at least 4 h in the dark. Finally, tissues were incubated at 85 °C in the oven until leaves reached a constant weight, and dry weight (DW) was measured. The relative water content (RWC) was calculated using the equation: RWC (%) = ((FW − DW)/(TW − DW)) × 100.

### 4.9. Statistical Analysis

The data were analyzed using a three-way analysis of variance (ANOVA), as implemented in the SPSS statistic package Version 17.0 (SPSS Inc., Chicago, IL, USA), and the differences between the means were compared using Tukey’s multiple range test (at a *p* < 0.05 significance threshold), with significant differences indicated by different letters above the bars in each figure. The data presented are means (± SD) of three independent experiments.

## 5. Conclusions

Drought stress during reproductive growth stages induced soybean seed weight to decrease through mechanisms that included drought stress regulating photosynthetic assimilate production, sugar metabolism, and transport in leaves and seeds. Drought stress inhibited the production of photosynthetic products, changed the distribution of photosynthetic products, and ultimately decreased the starch content of leaves. Drought stress enhanced the utilization efficiency of sucrose by increasing the activity levels of sucrose synthesis and degradation enzymes and reduced the secretion of sucrose from mesophyll cells by inhibiting SWEET transport capacity in leaves. Together, these mechanisms increased the content of sucrose in leaves in response to drought stress. At the beginning of seed formation, drought stress up-regulated the expression levels of *GmSUC2*, *GmSWEET12*, and *GmSWEET21* in seeds to promote more sucrose in seeds and increase the activities of sucrose synthesis and degradation enzymes to enhance utilization efficiency of sucrose in seeds. This enabled soybean plants to reserve energy and better tolerate drought stress. However, during the late seed filling stages, drought stress seriously weakened the capacity of sucrose transport from leaves to seeds and inhibited the degradation and circulation of sucrose, which was accompanied by a decreased hexose-to-sucrose ratio in seeds, together resulting in seed weight loss. In short, drought stress disrupted the balance of sucrose metabolism and transport in leaves and seeds at reproductive stages, which seemed to be the main mechanism through which drought caused soybean seed weight to decrease.

## Supplementary Materials

Supplementary materials can be found at https://www.mdpi.com/1422-0067/21/2/618/s1.

## Abbreviations

SPS	Sucrose phosphate synthase
UDPG	Uridine diphosphate glucose
SuSy	Sucrose synthase
NI	Cytoplasmic neutral/alkaline invertase
AI	Acid invertase
SWEETs	SWEETs transporter family
SUC	Sucrose transporter
ABA	Abscisic acid
*P*_N_	Net photosynthesis rate
CV.SN17	Shennong17 cultivar
CV.SN8	Shennong8 cultivar
CV.SN12	Shennong12 cultivar
DAF	Day after flowering
G	Genotype
S	Plant growth stage
T	Treatment
CWIN	Cell wall invertase
INV	Soluble invertase
CIN	Neutral invertase
GmEF1a	Translation elongation factor 1α
GmEF1b	Translation elongation factor 1β
