# Supplementary material for: Effect of Drought Stress during Soybean R2–R6 Growth Stages on Sucrose Metabolism in Leaf and Seed
Source: Int J Mol Sci. 2020 Jan 17;21(2):618. doi: 10.3390/ijms21020618 (PMC7013680; doi:10.3390/ijms21020618)
Supplement: Supplementary file 1 [file ijms-21-00618-s001.zip › ijms-693841-SI.docx]

Additional File 1: Supplementary Data

Supplementary Material

Effect of drought stress during soybean R2-R6 growth stages on sucrose metabolism in leaf and seed

Yanli Du, Qiang Zhao, Liru Chen, Xingdong Yao, Huijun Zhang, Junjiang Wu, Futi Xie*

* Correspondence: Futi Xie: xft299@syau.edu.cn

## Supplementary Figure

**Figure S1. Effect of drought stress on the relative water content (RWC) (%) of soybean leaves. Standard deviations were calculated with three independent experiments each comprising two soybean plants. Different letters above vertical bars indicate significant differences between means at a *P* < 0.05 level.**

**Figure S2. Effect of drought stress on hexose-to-sucrose ratio in (A) leaves and (B) seeds. Standard deviations were calculated with three independent experiments each comprising two soybean plants. Different letters above vertical bars indicate significant differences between means at a *P* < 0.05 level.**

**Figure S3. Relative expression levels of all selected genes in leaf (A) and seed (B) of CV.SN12. The genes expression levels were calculated according to the 2^-△t^ method.**

**Figure S4. Light response curves of photosynthesis. The light saturation point (LSP) was 1191** **μmol·m^-2^·s^-1^. The light compensation point (LCP) was 54 μmol·m^-2^·s^-1^. The respiration (Rd) was -3.15 μmol·m^-2^·s^-1^. The apparent quantum rate (AQE) was 0.057.**

## Supplementary Table

**Table S1. Analysis of variance in *P*_N_, shoot biomass, sugar contents, and sucrose metabolism-related parameters in shoots of three soybean cultivars.**

**Table S2. Analysis of variance in seed weight, sugar contents, and sucrose metabolism-related parameters in the seeds of three soybean cultivars.**

**Table S3. Specific primers used in this study**





**Figure S1. Effect of drought stress on the relative water content (RWC) (%) of soybean leaves. Standard deviations were calculated with three independent experiments each comprising two soybean plants. Different letters above vertical bars indicate significant differences between means at a *P* < 0.05 level.**


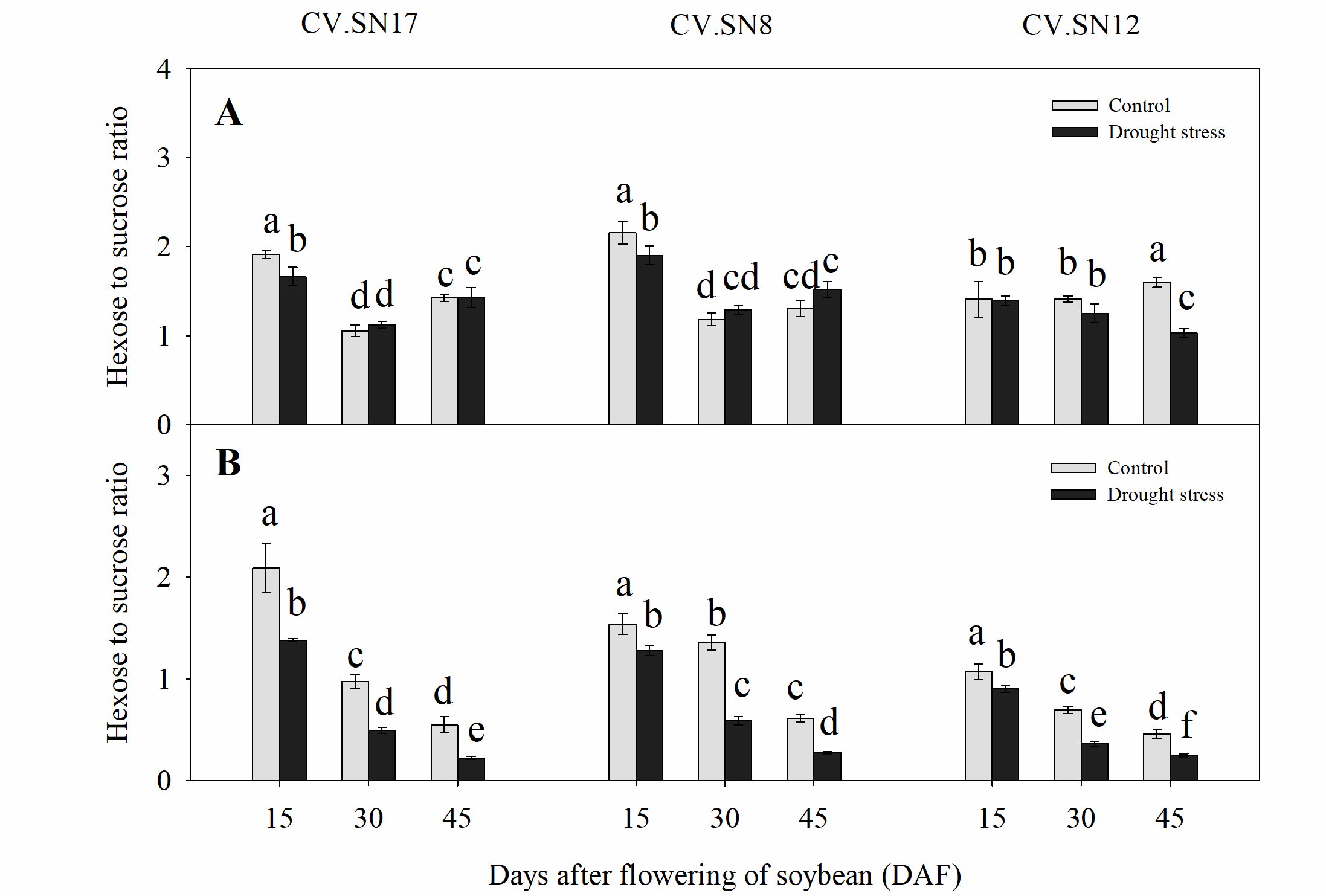


**Figure S2. Effect of drought stress on hexose-to-sucrose ratio in (A) leaves and (B) seeds. Standard deviations were calculated with three independent experiments each comprising two soybean plants. Different letters above vertical bars indicate significant differences between means at a *P* < 0.05 level.**

**

**

**Figure S3. Relative expression levels of all selected genes in leaf (A) and seed (B) of CV.SN12. The genes expression levels were calculated according to the 2^-△t^ method.**





**Figure S4. Light response curves of photosynthesis. The light saturation point (LSP) was 1191 μmol·m^-2^·s^-1^. The light compensation point (LCP) was 54 μmol·m^-2^·s^-1^. The respiration (Rd) was -3.15 μmol·m^-2^·s^-1^. The apparent quantum rate (AQE) was 0.057.**

**Table S1.** Analysis of variance in *P*_N_, shoot biomass, sugar contents, and sucrose metabolism-related parameters in shoots of three soybean cultivars.

| **Traits (/Plant)** | **Source of Variations** | | | | | | |
| --- | --- | --- | --- | --- | --- | --- | --- |
|  | **Growth Stage (S) (df = 2)** | **Genotype**  **(G) (df = 2)** | **Treatment (T) (df = 1)** | **S×G**  **(df = 4)** | **S×T**  **(df = 2)** | **G×T**  **(df = 2)** | **S×G×T**  **(df = 4)** |
| *P*_N_ [μmol(CO2) m^–2^ s^–1^] | ** | ** | ** | ** | ** | ** | ** |
| shoot biomass (g) | ** | NS | ** | ** | ** | ** | ** |
| Starch (mg g^-1^) | ** | ** | ** | NS | ** | ** | * |
| Sucrose (mg g^-1^) | ** | ** | ** | ** | ** | ** | ** |
| Fructose (mg g^-1^) | ** | ** | ** | ** | ** | ** | ** |
| Glucose (mg g^-1^) | ** | ** | ** | ** | NS | NS | NS |
| SPS (mmol sucrose mg^-1^ protein h^-1^) | ** | ** | ** | ** | ** | * | NS |
| SuSy (mmol glucose mg^-1^ protein h^-1^) | ** | ** | ** | ** | ** | NS | * |
| AI (mmol glucose mg^-1^ protein h^-1^) | ** | ** | ** | ** | ** | ** | ** |
| NI (mmol glucose mg^-1^ protein h^-1^) | ** | ** | NS | ** | * | ** | ** |
| *GmSUC2* | ** | ** | ** | ** | ** | NS | ** |
| *GmSWEET6* | ** | ** | ** | ** | ** | ** | ** |
| *GmSWEET15* | ** | ** | ** | ** | ** | ** | ** |
| *GmSPS1* | ** | ** | ** | ** | ** | ** | ** |
| *GmSPS2* | ** | ** | ** | ** | ** | ** | ** |
| *GmSPS3* | ** | ** | ** | ** | ** | ** | ** |
| *GmSPS4* | ** | ** | ** | ** | ** | ** | ** |
| *GmSuSy1* | ** | ** | ** | ** | ** | ** | ** |
| *GmSuSy2* | ** | ** | ** | ** | ** | ** | ** |
| *GmC-INV* | ** | ** | ** | ** | ** | ** | ** |
| *GmA-INV* | ** | ** | ** | ** | ** | ** | ** |

* and ** indicate significance at the 0.05 and 0.01 probability levels, respectively. NS, Not significant.

**Table S2.** Analysis of variance in seed weight, sugar contents, and sucrose metabolism-related parameters in the seeds of three soybean cultivars.

| **Traits (/Plant)** | **Source of Variations** | | | | | | |
| --- | --- | --- | --- | --- | --- | --- | --- |
|  | **Growth Stage (S) (df = 2)** | **Genotype (G)**  **(df = 2)** | **Treatment (T)(df = 1)** | **S×G**  **(df = 4)** | **S×T**  **(df = 2)** | **G×T**  **(df = 2)** | **S×G×T**  **(df = 4)** |
| Seed weight (g) | ** | ** | ** | ** | ** | ** | ** |
| Starch (mg g^-1^) | ** | * | ** | ** | ** | ** | ** |
| Sucrose (mg g^-1^) | ** | ** | ** | * | ** | ** | ** |
| Fructose (mg g^-1^) | ** | ** | ** | ** | ** | ** | ** |
| Glucose (mg g^-1^) | ** | ** | ** | ** | ** | ** | ** |
| SPS (mmol sucrose mg^-1^ protein h^-1^) | ** | ** | ** | ** | ** | NS | ** |
| SuSy (mmol glucose mg^-1^ protein h^-1^) | ** | ** | ** | ** | ** | ** | ** |
| AI (mmol glucose mg^-1^ protein h^-1^) | NS | ** | ** | NS | ** | ** | ** |
| NI (mmol glucose mg^-1^ protein h^-1^) | ** | ** | ** | ** | ** | ** | ** |
| *GmSUC2* | ** | ** | ** | ** | ** | ** | ** |
| *GmSWEET12* | ** | ** | ** | ** | ** | ** | ** |
| *GmSWEET21* | ** | ** | ** | ** | ** | ** | ** |
| *GmSPS1* | ** | ** | ** | ** | ** | ** | ** |
| *GmSPS2* | ** | ** | ** | ** | ** | ** | ** |
| *GmSPS3* | ** | ** | ** | ** | ** | ** | ** |
| *GmSPS4* | ** | ** | NS | ** | ** | ** | ** |
| *GmSuSy1* | ** | ** | ** | ** | ** | NS | ** |
| *GmSuSy2* | ** | ** | ** | ** | ** | ** | ** |
| *GmC-INV* | ** | ** | ** | ** | ** | ** | ** |
| *GmA-INV* | ** | ** | ** | ** | ** | ** | ** |

* and ** indicate significance at the 0.05 and 0.01 probability levels, respectively. NS, Not significant.

**Table S3. Specific primers used in this study**

| Gene Name | Primer ID | Primer sequence (5’-3’) |
| --- | --- | --- |
| GmSPS1 | Glyma.17G109700_F | AAGCAGCTGGAGAGTGAGACAG |
|  | Glyma.17G109700_R | TCCGACAAGTCCTCAGACATATCC |
| GmSPS2 | Glyma.14G029100_F | GGCACTCAGGTATCTCTTTGTACG |
|  | Glyma.14G029100_R | AGGATGACGTACATGTTTGCAACG |
| GmSPS3 | Glyma.08G308600_F | AGAGGGTAACAAGCCGAAGATTGG |
|  | Glyma.08G308600_R | ATGTCCTCTGTTGCCTCTCTGC |
| GmSPS4 | Glyma.06G323700_F | TGTGAAATCAGGAGCCATGATCCG |
|  | Glyma.06G323700_R | AGCGTAAGCCTCTCATTCGCAAC |
| GmSuSy1 | Glyma.13G114000_F | AGAAGTGCAAGCTTGACCCAACTC |
|  | Glyma.13G114000_R | ATACGCTGGAGACCAGCCTTTG |
| GmSuSy2 | Glyma.03G216300_F | ATCCTCAAGTTCCGTGATTTGGC |
|  | Glyma.03G216300_R | TGTCTGCTGTCAAGCCTTGGTG |
| GmA-INV | Glyma.05G056300_F | AAACCAGGGTCAGTGGTGTCAG |
|  | Glyma.05G056300_R | CTCGGCAACAATGTCCAACTGC |
| GmC-INV | Glyma.20G177200_F | CGTTGCCATCCTCCTTCAGATAG |
|  | Glyma.20G177200_R | AGGGATAGAGCAGTGAGAGCTG |
| GmSUC2 | Glyma.16G157100_F | GGCCAAGGTTTATCTTTGGGAGTC |
|  | Glyma.16G157100_R | CAAGTTGCCACCACCAAACAAAG |
| GmSWEET6 | Glyma.04G198600_F | GAAAGCGTTATTGCTCCAACAGTG |
|  | Glyma.04G198600_R | TCCTCAGGTTGTTGTTGATCTTGC |
| GmSWEET15 | Glyma.06G166800_F | CATGACCAAACGGAACTCAACAAG |
|  | Glyma.06G166800_R | AACTTCGCAACTCACCCTCTCC |
| GmSWEET12 | Glyma.05G202700_F | GTGAAGGCTCAAGAATTGAATGGC |
|  | Glyma.05G202700_R | AGTAGCTGCGTGATTCGGTTCC |
| GmSWEET21 | Glyma.08G010000_F | CTACCTGTCAAAGGGAGCAAAGCG |
|  | Glyma.08G010000_R | ACGCTCCTCGTCTTTATGACTCG |
| GmEF1a | Glyma.05G114900_F | GACCTTCTTCGTTTCTCGCA |
|  | Glyma.05G114900_R | CGAACCTCTCAATCACACGC |
| GmEF1b | Glyma.14G039100_F | GTTGAAAAGCCAGGGGACA |
|  | Glyma.14G039100_R | TCTTACCCCTTGAGCGTGG |
